# The early sport specialization paradox: an argument for early sport diversification in female athletes

**DOI:** 10.3389/fpsyg.2024.1438075

**Published:** 2024-07-31

**Authors:** Maya Beninteso

**Affiliations:** Department of Psychology, Simon Fraser University, Burnaby, BC, Canada

**Keywords:** female athlete, early sport specialization, early sport diversification, trauma, young athlete

Early sport specialization (ESS) has emerged as a contentious topic within the last two decades given its assertion that ESS is necessary for athletic achievement (Côté et al., 2020). However, not only has ESS been refuted as the sole pathway to athletic excellence, it is further associated with a number of trauma-related disorders and correlates thereof, such as: post-traumatic stress disorder, eating disorders and disordered eating, non-suicidal self injury, suicide, substance use, and more. Interestingly, the aforementioned trauma-related disorders are more pronounced in specialized, female athletes (Herrero et al., 2021; van Niekerk et al., 2023). This discrepancy in ESS and its effect on female athletes is concerning and calls for action. Early sport diversification (ESD) lacks the alarming associations with trauma-related disorders, providing a viable replacement for ESS. Accordingly, the purpose of the paper is to: (1) delineate unique experiences and considerations for female athletes; (2) characterize the nature of ESS; (3) denote specialization's association with trauma; and (4) describe and propose ESD as an alternative for ESS. I will assert that ESS is harmful—especially for female athletes—and that following the philosophy of ESD may prevent, or reduce, trauma associated with ESS in female athletes, whilst achieving its athletic-related intentions due to its emphasis on athlete development in lieu of obsessive athletic performance.

## Unique considerations for female athletes

Female athletes are a growing population that are neglected within the literature (Perry et al., 2021). The limited research suggests that, regardless of age, female athletes experience common mental disorders, such as: anxiety, depression, and eating disorders at a higher rate than their male counterparts (Herrero et al., 2021; van Niekerk et al., 2023). The aforementioned gendered discrepancy poses a unique question: what mechanisms are driving the pronounced association between female athletes and mental disorders? There are several, distinct factors that female athletes face as a result of their gender. For instance, Lunde and Gattario (2017) suggest that female athletes may experience a conflict between feminine beauty standards and the physical demands of their respective sports. There appears to be a constant struggle between “performance culture,” fueling one's body to optimally function as an athlete, and “appearance culture,” conforming to the female thin ideal (Lunde and Gattario, 2017). However, female athletes constantly lose this battle as they are regularly criticized for (1) possessing the ideal body for their sport and looking too masculine, or (2) possessing the ideal body for society and being deemed as weak (Lunde and Gattario, 2017). Moreover, Pascoe et al. (2022) delineate that female athletes are more likely to experience all forms of harassment and abuse, including: sexual, physical, psychological, and neglect. Recent developments in social media technology have been utilized by coaches to further perpetuate sexual violence against young female athletes, such as Snapchat and its disappearing photos and messages (Sanderson and Weathers, 2020). Additionally, the physiological and psychological health of female athletes may be compromised and complicated by disordered eating (Wolfenden et al., 2022; van Niekerk et al., 2023). Relative Energy Deficiency in Sport (RED-S)—formerly known as the Female Athlete Triad—is defined as the discrepancy between energy expended and a female athlete's nutritional intake (van Niekerk et al., 2023). As noted by Wolfenden et al. (2022), RED-S has further been known to be associated with anxiety and depression symptoms. While certainly not exhaustive, the aforementioned factors call for the further examination of female athletes and their experiences.

## Early sport specialization and correlates of trauma

Early sport specialization (ESS) is generally defined as the dedication to a singular sport prior to a certain age (Zampieri, 2024). However, there are definitional differences with respect to the age at which sport specialization is considered ESS and other training-related factors (Sugimoto et al., 2019). The rationale behind ESS includes beliefs surrounding the notion that earlier, specialized training equals various athletic successes, including: excellence in the sport of choice, athletic awards or scholarship, a professional athletic career, and/or competing in the Olympics (Jayanthi et al., 2013). Jayanthi et al. (2013) reinforced the prominence of the aforementioned beliefs, stating that the general consensus amongst experts was that early specialization was paramount to achieving athletic success.

Whilst the intentions of ESS are simply to reach athletic aspirations, there appears to be a disconnect between its objectives and reality—especially for female athletes. Female athletes subjected to ESS are associated with several trauma-related disorders and risk factors thereof, including: injuries, posttraumatic stress disorder (PTSD), eating disorders and disordered eating, non-suicidal self injury (NSSI), and substance use disorders.

### Post-traumatic stress disorder

Unsurprisingly, the prominence of PTSD amongst elite athletes is elevated compared to their non-athlete counterparts (Aron et al., 2019). Athletes are exposed to a number of unique potentially traumatic events, including: injuries, witnessing a traumatic event such as the injury of a teammate, and enduring abusive team dynamics (Aron et al., 2019). With respect to injuries, Padaki et al. (2018) explored PTSD post anterior cruciate ligament (ACL) rupture, examining correlates of: athletic identity, gender, and sport specialization on PTSD symptoms. It was asserted that female athletes, young athletes, and athletes with a strong athletic identity are at an elevated risk for PTSD symptoms post ACL-rupture (Padaki et al., 2018). Padaki et al. (2018) further found that athletes specializing in a singular sport possessed the highest association with high athletic identification. Additionally, Padaki et al. (2018) assert that several traits are associated with experiencing severe psychological trauma post ACL-rupture, including: female athletes, high school and collegiate athletes, and athletes with high athletic identification. The aforementioned findings are significant given that young, specialized female athletes possess the outlined risk factors associated with PTSD post ACL-rupture, thereby exacerbating their risk for the trauma-related disorder. Succinctly, they are the population with the most risk for experiencing increased PTSD symptoms post ACL rupture. Moreover, PTSD has been explored in the context of female athletes and their experiences with sexual violence in sport. Coaches and coaching staff are in a unique position of power to abuse their female athletes, including—but not limited to—sexual violence (Ohlert et al., 2020). Ohlert et al. (2020) suggest that female athletes experience more sexual violence than their male athlete counterparts, with a lifetime prevalence of sexual violence of two-thirds for elite female athletes. Although more cases of sexual assault occur outside the confines of sports, the lifetime prevalence of sexual violence for elite athletes remains higher than the general population (Ohlert et al., 2020). Aron et al. (2019) highlight the significance of sexual assault in female athlete populations, stating that nearly one-quarter of athletes diagnosed with PTSD have experienced sexual assault. The unique power structure of ESS, including coach access and control, leaves specialized female athletes at risk for sexual assault and subsequent PTSD. In sum, female athletes who have experienced ESS are at a significantly increased risk for PTSD than their male and female non-athlete counterparts.

### Eating disorders and disordered eating

Eating disorders (EDs) and disordered eating (DE) are especially prominent amongst female athletes (Martinsen and Sundgot-Borgen, 2013). Martinsen and Sundgot-Borgen (2013) highlight that elite female athletes are at the most risk, possessing higher incidences of EDs than their female non-athlete and male athlete counterparts. However, said risk is most pronounced for elite female athletes in body-focused sports, such as gymnastics, figure skating, and weight-class sports (Blagrove et al., 2017). This is intuitive given that the previously mentioned “appearance culture” and “performance culture” are harmonious in their body standards for body-focused sports—both emphasize a thin ideal (Blagrove et al., 2017; Lunde and Gattario, 2017). In addition to physiological disruptions associated with EDs and DE, they are further correlated with suicide and NSSI (Lash, 2013; Blagrove et al., 2017). In general, over 20% of deaths associated with anorexia nervosa (AN) are due to suicide (Joy et al., 2016). However, specific research on athletes with EDs suggest that they are 5% more likely to attempt suicide than the general population (Blagrove et al., 2017). Lash (2013) further outlines a disturbing association between DE and NSSI, finding that DE is highly correlated with NSSI behaviors. Although suicides are greater amongst retired athletes than active athletes generally, the unique intersections of being a specialized athlete and a woman pose several intersectional concerns (Pichler et al., 2023). Succinctly, EDs and DE are prominent concerns for specialized female athletes given the disturbing correlates with which they are associated.

### Injuries

Injuries are a unique experience—and risk factor—associated with ESS and female athletes (Herrero et al., 2021). Numerous domains of injuries have been explored in the literature, such as: concussions, overuse injuries, and injuries requiring surgical intervention. Unsurprisingly, the aforementioned injuries and injury interventions are all associated with trauma or trauma-related disorders. Generally, Cairo et al. (2022) assert that highly specialized female athletes possess staggering numbers of injuries. Namely, Okoruwa et al. (2022) found that highly specialized female athletes were at a greater risk for concussions and injuries than their moderately and low specialized counterparts. More notably, Wang et al. (2024) suggest that sport-related concussions are associated with increases in: suicidal ideation, suicide planning, suicidal attempt, and NSSI. This is alarming given the disproportionate rate of concussions for specialized female athletes (Okoruwa et al., 2022). Additionally, overuse injuries are prominent amongst specialized female athletes and are associated with several trauma and trauma-related concerns (Jayanthi and Dugas, 2017). Jayanthi and Dugas (2017) delineate that the risk of an overuse injury is associated with the degree of specialization, noting that female athletes are drawn to more specialized sports such as dance or tennis. However, as noted by Sugimoto et al. (2019) the increased prevalence of overuse injuries may be attributed to the training volume associated with specialized athletics. Some overuse injuries cannot subside without surgical intervention and there are severe psychological consequences associated with surgical intervention (Smith et al., 2013). Accordingly, Sweeney et al. (2021) suggest that a higher proportion of female collegiate athletes sustain injuries requiring surgical intervention, one of which are ACL ruptures. In fact, post ACL-rupture athletes were found to experience pronounced PTSD symptoms, such as: hyperarousal, intrusion, and avoidance (Padaki et al., 2018). Most notably, the aforementioned PTSD were the most pronounced in female athletes (Padaki et al., 2018). Daley et al. (2023) reiterated the mental health and trauma-related concerns associated with injury recovery in specialized athletes, including post-surgical intervention. Injury recovery is associated with increased risk for: PTSD, DE, substance use, depression, and anxiety (Daley et al., 2023).

## Early sport diversification

Wiersma (2000) defines early sport diversification (ESD) as the participation in multiple sports wherein the development in cognitive and physical skills increases due to cross-over between sports. For instance, a child who plays baseball may mobilize skills from other sports to improve their overall athletic performance (Caruso, 2013). Instead of the emphasis on training, ESD nurtures the notion of playing in lieu of the pressure of excelling in a single sport (Caruso, 2013).

Furthermore, ESD appears to achieve the athletic goals associated with ESS without its trauma-related correlates. Jayanthi et al. (2013) state that ESD's environment is associated with psychosocial, cognitive, and physical benefits—all of which promote motivation. Although ESS proposes that early, dedicated training to a single sport increases chances of athletic success, this does not appear to be the case. In fact, for the majority of sports, ESD increases athletic success, with most NCAA Division I athletes having been early diversifiers—not specializers (Jayanthi et al., 2013). Athletes who engaged in ESD and subsequently specialized later in their athletic careers reported: increased participation in sport, decreased injuries, and increased enjoyment in sport (Jayanthi et al., 2013). Ryder et al. (2021) posit that the aforementioned benefits associated with ESD were further replicated in female athlete population. This is notable given the association between female ESS, injuries, and trauma; if ESD is associated with decreased injuries, this could act as a buffer for young female athletes.

The concept of ESD is under-researched, especially for female athletes. The emphasis on the current literature is on comparing athletic success amongst early specializers and diversifiers. However, limited attention has been paid to explicit mental health and trauma impacts. Despite the lack of research, the limited literature points to a hope for the prevention or decrease in trauma-related disorders for young athletes.

## Counterargument

Despite the outlined trauma and trauma-related disorders associated with ESS, there are valid counterarguments in demonizing ESS and utilizing ESD. Firstly, Mosher et al. (2022) denote that little is known about the driving mechanism behind the association between ESS and trauma-related disorders. For this reason, it may be too early to demonize ESS itself. There may be a mediating variable associated, or co-occurring, with ESS that is resulting in an increased association with trauma-related disorders (Mosher et al., 2022). This begs the question as to whether there is an age, or set of conditions, wherein ESS does not increase the association between specialization and trauma. Moreover, whilst the literature has not focused on the potential downfalls of ESD, parental figures have voiced their practical concerns of sport diversification. As per Strashin (2018), many individuals understand the benefits of early diversification, however it may not be feasible. Firstly, parents cite the time and financial constraints associated with ESD, stating that sports are expensive and a parent/guardian must be available to take the young athlete to and from sporting events (Strashin, 2018; Kroshus et al., 2021). Intuitively, given the cost associated with multiple sports, individuals of lower socioeconomic status (SES) may not be able to afford sport diversification. This may leave their child in a single, specialized sport for said financial reason, potentially resulting in disproportionate levels of the outlined trauma-related disorders for lower SES individuals. In summary, not only has ESS yet to be identified as the causal variable associated with trauma, but ESD possesses several practical concerns for parental figures.

## Rebuttal

Although there are some counterarguments opposing the proposed harms of ESS and the feasibility of ESD, one may refute such concerns. Namely, in regards to a potential mediating variable driving the association between trauma ESS, it would be egregious to ignore the existing literature highlighting this association. Regardless of the driving mechanism, it would be reckless to expose a child to ESS merely because there has yet to be a causal link or identified factor associated with ESS. The potential mediating variable does not detract from the current research that denounces ESS due to its link with: injuries, PTSD, EDs, DE, and more. Conversely, with respect to the feasibility of ESD, Wiersma's (2000) definition of ESD did not explicitly include costly, competitive sports. This loose definition of engaging in multiple sports allows for recreational and competitive play (Wiersma, 2000). Accordingly, regular pick-up basketball games could be included under the definition of “multiple sports.” Even if the definition of ESD was exclusive to organized, competitive sports, there are still resources for low SES families to access sport. In fact, Hernandez et al. (2023) conducted interviews with low SES parental figures, finding that there are resources to decrease the financial barrier, such as: lower cost leagues, lower cost competitive teams, lower cost programs, and youth athletic scholarships or grants. In sum, participation in sport neither has to be organized, nor expensive. There are several programs and sources of funding available to low SES athletes and youth so that they may access athletics (Hernandez et al., 2023).

## Discussion

ESS remains a contentious topic with a set of intentions that do not align with reality. In practice, ESS does not increase athletic success despite the belief that early, dedicated training translates to athletic achievement (Jayanthi et al., 2013). Even so, there are far too many associations between trauma-related disorders and ESS. Despite research asserting the benefits of sports and physical activity generally, ESS is associated with a number of trauma-related disorders and mental health challenges that are pronounced in female populations (Martinsen and Sundgot-Borgen, 2013; Padaki et al., 2018; Aron et al., 2019). Furthermore, ESS may compromise an athlete's health with the physiological and psychological ramifications associated with injury and injury intervention (Padaki et al., 2018; Cairo et al., 2022; Okoruwa et al., 2022; Daley et al., 2023; Wang et al., 2024). Given the evidence countering ESS as a means to achieve athletic success and its association with trauma, ESS appears to be an unsafe and ineffective way to reach athletic aspirations. However, ESD remains an—albeit underresearched—but promising alternative to ESS. ESD is associated with preliminary benefits, including achieving athletic excellence and general sports enjoyment (Jayanthi et al., 2013). Future research should focus on whether ESD is associated with less instances of trauma-related disorders.

Clinical implications in the treatment of specialized female athletes may further be applied in light of the aforementioned findings. Given the prominence of trauma-related disorders within said population, clinicians must further screen for the outlined disorders in order for early treatment and prevention of further mental health challenges (Aron et al., 2019). Aron et al. (2019) posit that screening of trauma-related disorders may be implemented as a prevention and identification tool within elite female sport settings. In doing so, female athletes may mobilize resources and obtain the care they require to thrive.

Despite the proposed alternative to ESS, this paper possesses several limitations. Primarily, the samples of the articles utilized were predominantly White participants. Thus, we do not know the true role of intersectionality within this area of sports psychology. Lastly, the definition of ESS varies widely from study to study; as such, there remains a question whether specialization is more or less harmful for a certain age group. With that said, more research is required on: the specific effects of ESD for female athletes, the factor(s) mediating the relationship between ESS and trauma-related disorders, and the effects of ESS on other intersectional identities/minority populations.

From championing for women to gain access to sports, to advocating for more research and subsequent policy implications, female athletes have remained unlike researchers within the field—on the ball.

## Author contributions

MB: Writing – original draft, Writing – review & editing.
